# Hollow MnO_2_ as a tumor-microenvironment-responsive biodegradable nano-platform for combination therapy favoring antitumor immune responses

**DOI:** 10.1038/s41467-017-01050-0

**Published:** 2017-10-12

**Authors:** Guangbao Yang, Ligeng Xu, Yu Chao, Jun Xu, Xiaoqi Sun, Yifan Wu, Rui Peng, Zhuang Liu

**Affiliations:** Institute of Functional Nano & Soft Materials (FUNSOM), Jiangsu Key Laboratory for Carbon-Based Functional Materials & Devices, Soochow University, 199 Ren’ai Road, Suzhou, 215123 Jiangsu China

## Abstract

Herein, an intelligent biodegradable hollow manganese dioxide (H-MnO_2_) nano-platform is developed for not only tumor microenvironment (TME)-specific imaging and on-demand drug release, but also modulation of hypoxic TME to enhance cancer therapy, resulting in comprehensive effects favoring anti-tumor immune responses. With hollow structures, H-MnO_2_ nanoshells post modification with polyethylene glycol (PEG) could be co-loaded with a photodynamic agent chlorine e6 (Ce6), and a chemotherapy drug doxorubicin (DOX). The obtained H-MnO_2_-PEG/C&D would be dissociated under reduced pH within TME to release loaded therapeutic molecules, and in the meantime induce decomposition of tumor endogenous H_2_O_2_ to relieve tumor hypoxia. As a result, a remarkable in vivo synergistic therapeutic effect is achieved through the combined chemo-photodynamic therapy, which simultaneously triggers a series of anti-tumor immune responses. Its further combination with checkpoint-blockade therapy would lead to inhibition of tumors at distant sites, promising for tumor metastasis treatment.

## Abstract (web summary)

MnO_2_ nanostructures are promising TME-responsive theranostic agents in cancer. Here, the authors develop a nano-platform based on hollow H-MnO_2_ nanoshells able to modulate the tissue microenvironment, release a drug and inhibit tumor growth alone or in combination with check-point blockade therapy.

## Introduction

The tumor microenvironment (TME), which is often featured with vascular abnormalities, high lactate levels, glucose deprivation, low pH values, and hypoxia^1–5^, is an important factor that largely affects the therapeutic outcomes in many conventional cancer therapies^6–11^. Moreover, it also been discovered that the unique features of TME may greatly limit the killing functions of cytotoxic T lymphocytes and promote the immunosuppression by multiple kinds of cells like myeloid-derived suppressor cells (MDSC), M2 tumor-associated macrophages (TAM), and regulatory T cells (Treg) within tumors, all of which are unfavorable for cancer treatment^12, 13^. Hence, designing nanoscale drug delivery systems (nano-DDSs) that are able to be responsive to the inherent features of TME has been proposed to be a promising approach to realized tumor-specific cancer treatment^14–16^. For instance, many smart nano-DDSs are able to release their payloads, or show reduced sizes/converted charges under TME conditions (e.g., reduced pH, hypoxia, tumor-specific enzymes, etc.), so as to realize improved therapeutic specificity and efficacy^17–19^. On the other hand, it has been proposed that by modulating the TME inside solid tumors, the therapeutic responses of those tumors to various types of cancer therapies may be significantly enhanced^20–22^. Therefore, designing TME-responsive and TME-modulating nano-DDSs may be of great interests for new generations of cancer combination therapies.

In recent years, MnO_2_ nanostructures have attracted substantial attention as a unique type of TME-responsive theranostic agents^23, 24^. It has been found that MnO_2_ nanostructures would be decomposed by reaction with either H^+^ or glutathione (GSH) existing within the TME, generating Mn^2+^ ions that are able to significantly enhance T1-magnetic resonance (MR) imaging contrast for tumor-specific imaging and detection^25–28^. Meanwhile, MnO_2_ nanostructures are able to trigger the decomposition of H_2_O_2_ existing in the TME into water and oxygen, so as to relieve tumor hypoxia^29–31^. Such an effect has been found to be able to enhance a number of cancer therapies such as radiotherapy and photodynamic therapy (PDT) in which oxygen is actively involved in the treatment process^23, 28, 29, 32, 33^. Moreover, as MnO_2_ nanoparticles could be decomposed to harmless water-soluble Mn^2+^ ions that are rapidly excreted by kidneys, there should be no long-term toxicity concerns for MnO_2_ nanostructures when they are used for in vivo applications, unlike many other non-biodegradable inorganic nanomaterials^23, 29^. However, most of previously reported MnO_2_ nanostructures are nanoparticles, nanosheets, or nanocomposites incorporated with other types of nanoparticles, and may not be ideal to realize the most effective drug loading as well as precisely controlled release of therapeutic payloads^32, 34^. Hollow nanostructures with mesoporous shells (e.g., hollow mesoporous silica) and large cavities have been demonstrated to be excellent drug loading/delivery systems to load high quantities of therapeutic agents, whose release may be precisely controlled by tuning the shell structures or coatings^35, 36^. However, hollow MnO_2_ nanostructures as smart DDs have not yet been reported to our best knowledge.

Herein, we therefore for the first time design an intelligent theranostic platform based on hollow mesoporous MnO_2_ (H-MnO_2_) nanoshells for tumor-targeted drug delivery, ultrasensitive pH-triggered controllable release, and TME-responsive generation of oxygen to overcome tumor hypoxia, so as to achieve tumor-specific enhanced combination therapy under the guidance of pH-responsive MR imaging. In this system, mesoporous MnO_2_ shells were grown on silica nanoparticles, which were then removed by gentle etching. The obtained H-MnO_2_ with hollow structures are functionalized with polyethylene glycol (PEG), yielding H-MnO_2_-PEG nanoparticle with great physiological stability. A photosensitizer, chlorine e6 (Ce6), and an anti-cancer drug, doxorubicin (DOX), can be co-loaded into this hollow H-MnO_2_-PEG nano-platform with high loading capacities (H-MnO_2_-PEG/C&D). Under acidic pH, the fast break-up of MnO_2_ nanoshells would lead to release of loaded drugs, and simultaneously result in significantly enhanced T1-contrast under MR imaging. After systemic injection of H-MnO_2_-PEG/C&D into tumor-bearing mice, strong Ce6 fluorescence and T1-weighted MR signals appear in both tumors and kidneys, suggesting efficient passive tumor homing of those nanoparticles, as well as their rapid renal filtration after being decomposed. Meanwhile, owing to MnO_2_-triggered in-situ generation of oxygen from tumor endogenous H_2_O_2_, the tumor oxygenation level is greatly improved. Great in vivo synergistic therapeutic effect is then achieved after combining chemotherapy and PDT with H-MnO_2_-PEG/C&D by a single treatment using a rather low dose. In addition, it is further discovered that combination therapy with this novel agent also could effectively induce anti-tumor immunities, which with the help of programmed death-ligand 1 (PD-L1) checkpoint blockade^37^ could inhibit tumor growth at distant sites spared from light exposure via a remarkable abscopal effect. Our work highlights the great promise of modulating TME with smart nano-systems to enhance the efficacies of various types of therapies to achieve a comprehensive effect in fighting cancers.

## Results

### Synthesis and characterization of H-MnO_2_-PEG

**Fig. 1:**
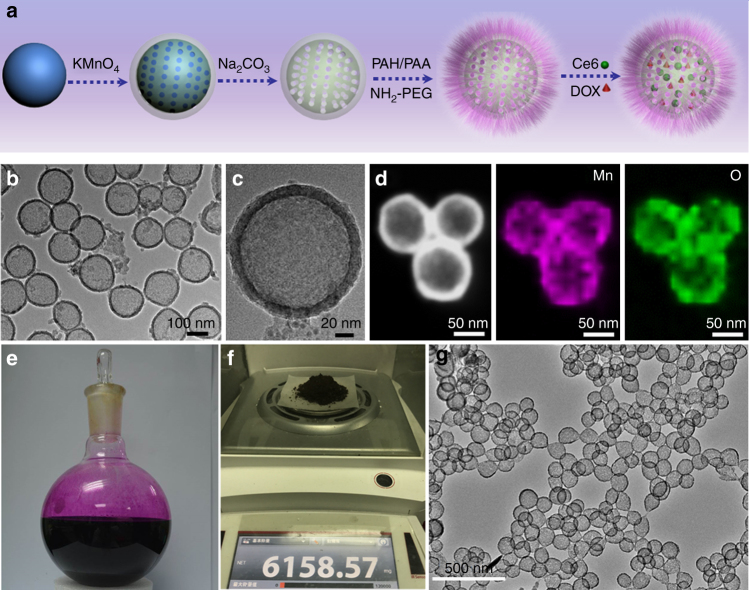
Synthesis and characterization of H-MnO_2_-PEG. **a** A scheme indicating the step-by-step synthesis of H-MnO_2_-PEG nanoparticles and the subsequent dual-drug loading. **b**, **c** A TEM image (**b**) and a magnified TEM image (**c**) of H-MnO_2_-PEG. **d** HAADF-STEM image and elemental mapping for H-MnO_2_-PEG. **e** A photo of the 500 mL reaction vessel for preparation of H-MnO_2_ in a large scale. **f** Digital picture showing the gram-scale production of H-MnO_2_ nanoshells in one reaction. **g** A representative TEM image of H-MnO_2_ nanoshells prepared in the gram-scale

The procedure for the synthesis of H-MnO_2_-PEG/C&D is illustrated in Fig. 1a. Firstly, monodispersed silica nanoparticles were synthesized by hydrolyzation of tetraethyl orthosilicate (TEOS) and then utilized immediately as the hard template. A uniform layer of mesoporous MnO_2_ was grown on the surface of as-made silica nanoparticles by simply mixing them with manganese permanganate (KMnO_4_), which was reduced by unreacted organosilica existing on those freshly prepared silica nanoparticles. The hollow mesoporous MnO_2_ (H-MnO_2_) nanoshells were obtained after incubating MnO_2_@SiO_2_ nanoparticles with a Na_2_CO_3_ solution to dissolve silica. To enhance their water solubility and physiological stability, H-MnO_2_ nanoshells were modified with PEG through a layer-by-layer (LBL) polymer-coating method. In this process, as-made H-MnO_2_ nanoshells with negative charges were coated subsequently with a cationic polymer poly (allylamine hydrochloride) (PAH), and then an anionic polymer poly (acrylic acid) (PAA) through electrostatic interactions. Amino-terminated PEG (NH_2_-PEG) was then conjugated to the surface of PAA-coated H-MnO_2_ nanoshells via amide formation, producing H-MnO_2_-PEG nanoshells. Lastly, the photosensitizer chlorine e6 (Ce6) and anti-cancer drug doxorubicin (DOX) were simultaneously loaded into the hollow structure of H-MnO_2_-PEG nanoshells, yielding H-MnO_2_-PEG/C&D, which was used for further experiments.

Transmission electron microscope (TEM) images of H-MnO_2_-PEG nanoshells clearly revealed the spherical morphology and the hollow structure of our product (Fig. 1b). The thickness of such MnO_2_ shell was measured to be ~15 nm (Fig. 1c). The hollow structure of H-MnO_2_-PEG nanoshells was further confirmed by the high-angle annular dark-field scanning TEM (HHAADF-STEM)-based elemental mapping (Fig. 1d). In the process of surface functionalization, the step-wise altered zeta potentials indicated successful LBL coating of polymers on those nanoparticles (Supplementary Fig. 1). With surface PEG coating, H-MnO_2_-PEG could be dispersed in different physiological buffers without any aggregation over time (Supplementary Fig. 2). Such a strategy could be scaled-up for gram-scale production of H-MnO_2_ nanoshells with high quality in one reaction batch (Fig. 1e–g).

### pH-dependent nanoparticle decomposition and drug behaviors

**Fig. 2:**
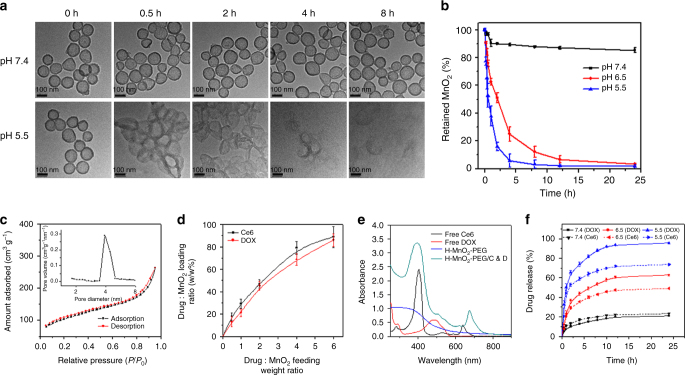
pH-dependent nanoparticle decomposition and drug behaviors of H-MnO_2_-PEG/C&D. **a** TEM images of H-MnO_2_-PEG after incubation in buffers with different pHs (7.4 and 5.5) for various periods of time. **b** The degradation behavior of H-MnO_2_-PEG dispersed in different pH values (7.4, 6.5 and 5.5) determined by the absorbance of MnO_2_. **c** Pore-size distribution curve and N_2_ adsorption/desorption isotherms (inset) of the H-MnO_2_-PEG sample. **d** Ce6 and DOX-loading weight ratios in H-MnO_2_-PEG/C&D at different feeding drug: MnO_2_ ratios. **e** UV–vis-NIR spectra of free Ce6, DOX, H-MnO_2_-PEG, and H-MnO_2_-PEG/C&D. **f** Percentages of released Ce6 and DOX from H-MnO_2_-PEG/C&D over time in the presence of 10% fetal bovine serum (FBS) at different pH values (7.4, 6.5, and 5.5). Date are presented as means ± standard deviation (s.d.) (*n* = 3)

Manganese dioxide (MnO_2_) is known to be stable under neutral and basic pH, but can be decomposed into Mn^2+^ under reduced pH values^38^. Therefore, TEM images of H-MnO_2_-PEG after incubation in phosphate-buffered saline (PBS) with different pH values (7.4 and 5.5) for various treated time were recorded (Fig. 2a). The morphology of H-MnO_2_-PEG nanoshells showed no significant change in pH 7.4 solution after 8 h, indicating that H-MnO_2_-PEG nanoshells were stable in the neutral environment. However, H-MnO_2_-PEG exhibited time-dependent degradation behavior in acidic solutions due to the decomposition of MnO_2_ into Mn^2+^ ions. The degradation rates could be determined by the decrease of MnO_2_-characteristic absorbance band (Fig. 2b; Supplementary Fig. 3), which appeared to be stable under pH 7.4 but decreased rapidly under pH 6.5 and 5.5, further demonstrating the ultrasensitive pH-responsive degradation behavior of H-MnO_2_-PEG.

The surface area and average pore diameter of H-MnO_2_-PEG were determined to be 360 m^2^ g^−1^ and 3.94 nm, respectively, by Brunauer–Emmett–Teller (BET) measurement (Fig. 2c). The hollow structure of H-MnO_2_-PEG with mesoporous shells is expected to be ideal for efficient drug loading. To employ MnO_2_ nanoshells for PDT application, the photosensitizer Ce6 was loaded into H-MnO_2_-PEG by incubating those nanoshells with different concentrations of free Ce6 under ultrasonication and stirring at room temperature. After removal of excess Ce6, UV–vis spectra were recorded to determine the Ce6-loading capacities. At the feeding weight ratio (Ce6: MnO_2_) of 3:1, the Ce6 loading researched a rather high ratio of 88.9% (Ce6: MnO_2_) (Fig. 2d). In this system, DOX, a commonly anti-cancer drug was also loaded into the hollow structure of nanoshells. For DOX loading, H-MnO_2_-PEG was mixed with different concentrations of DOX in dark for 12 h. The DOX loading increased with increasing added DOX and achieved the maximal level at 86.1%. Lastly, Ce6 and DOX also could simultaneously loaded into the hollow structure of H-MnO_2_-PEG nanoshells, obtaining dual-drug co-loaded H-MnO_2_-PEG/C&D nanoparticles for combination therapy (Fig. 2e).

The drug release behaviors of Ce6 and DOX from H-MnO_2_-PEG/C&D were then studied in solutions at different pH values (Fig. 2f). Compared with the slow drug-release profiles of H-MnO_2_-PEG/C&D at pH 7.4, the release speeds of both Ce6 and DOX were found to be much faster in mild acidic solutions at pH 6.5 and pH 5.5, owing to the acidic triggered decomposition of H-MnO_2_ nanocarriers into Mn^2+^ ions.

### In vitro experiments with H-MnO_2_-PEG/C&D

**Fig. 3:**
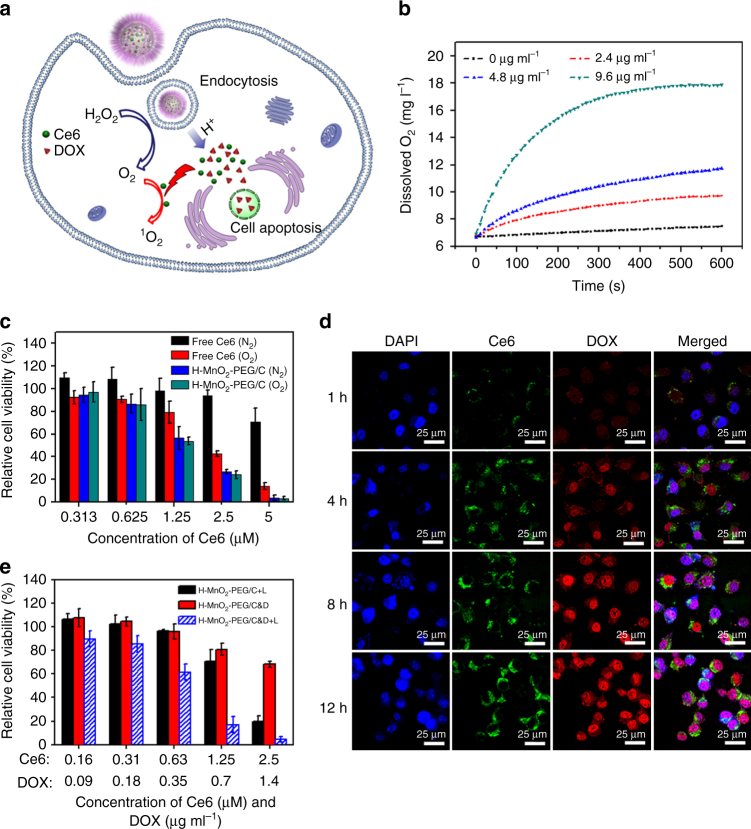
In vitro experiments with H-MnO_2_-PEG/C&D. **a** A scheme illustration of H-MnO_2_-PEG/C&D for pH-responsive drug delivery and oxygen-elevated PDT. **b** The O_2_ concentration changes in H_2_O_2_ solutions (100 μM) after various concentrations of H-MnO_2_-PEG/C&D were added. **c** In vitro PDT treatment of 4T1 cells by free Ce6 or H-MnO_2_-PEG/C under 660-nm light irradiation (5 mW cm^−2^ for 30 min) in N_2_ or O_2_ atmospheres. **d** Confocal images of 4T1 cells treated with H-MnO_2_-PEG/C&D at different times points. Blue, green, and red represent DAPI, Ce6, and DOX fluorescence, respectively. **e** Relative viabilities of 4T1 cells after incubation with H-MnO_2_-PEG/C with light irradiation, or H-MnO_2_-PEG/C&D with or without 660-nm light irradiation (5 mW cm^−2^, 30 min). Date are presented as means ± s.d. (*n* = 5)

As uncovered in many previous studies, the hypoxic TME is responsible for the limited PDT efficacy for treatment of solid tumors as oxygen is an essential element in the process of PDT^39, 40^. Considering the existence of endogenous H_2_O_2_ with concentrations in the range of 10–100 μM inside most types of solid tumors^41^, we then tested the ability of our H-MnO_2_ as a catalyst to trigger the decomposition of H_2_O_2_ by using an oxygen probe to measure the dissolved O_2_ after different concentrations of H-MnO_2_-PEG were added into H_2_O_2_ solutions (100 μM). Although the dissolved O_2_ level was stable in the H_2_O_2_ solution without adding H-MnO_2_-PEG nanoshells, we found that H-MnO_2_-PEG could effectively trigger the fast production of O_2_ from H_2_O_2_ by a MnO_2_ concentration-dependent manner (Fig. 3b).

The singlet oxygen (SO) generated from Ce6 and H-MnO_2_-PEG/C under 660-nm light irradiation in the presence of H_2_O_2_ was then monitored by a SO sensor green (SOSG) probe (Supplementary Fig. 4). As expected, no appreciable difference in SO production by free Ce6 under light exposure was observed regardless of H_2_O_2_ addition (100 μM). Interestingly, after addition of H_2_O_2_, the light-triggered SO production of H-MnO_2_-PEG/C was remarkably accelerated. Therefore, Ce6-loaded H-MnO_2_-PEG is expected to be a more effective PDT agent under TME conditions with low oxygen and high H_2_O_2_ contents^23^.

Next, we ought to study the efficacy of H-MnO_2_-PEG as a multifunctional DDS at the in vitro level (Fig. 3a). We firstly tested the cytotoxicity of H-MnO_2_-PEG in the dark (Supplementary Fig. 5). The standard methyl thiazolyl tetrazolium (MTT) assay indicated that H-MnO_2_-PEG exhibited no obvious toxicity to 4T1 murine breast cancer cells even at high concentrations of H-MnO_2_ up to 100 μg mL^−1^.

To evaluate the PDT efficiency of Ce6-loaded H-MnO_2_-PEG, 4T1 cells were incubated with H-MnO_2_-PEG/C or free Ce6 in either N_2_ or O_2_ environment for 2 h. Those cells were then treated with 660 nm light (5 mW cm^−2^, 30 min). Twenty-four hours later, their relative viabilities were determined by the MTT assay (Fig. 3c). Both free Ce6 and H-MnO_2_-PEG/C showed high photo-toxicity to cells within the oxygen atmosphere. In contrast, although the PDT-induced cell killing by free Ce6 under the nitrogen atmosphere was found to be much less effective, the light-triggered cancer cell-destruction efficiency of H-MnO_2_-PEG/C remained at high levels even under this hypoxic condition, likely owing to the additional supply of oxygen by MnO_2_-trigged decomposition of H_2_O_2_ generated in situ by cancer cells. Therefore, different from conventional PDT, which is effective only under normoxic conditions, Ce6-loaded H-MnO_2_-PEG could serve as an effective PDT agent even within the hypoxic environment.

We next used DOX and Ce6 co-loaded H-MnO_2_-PEG (H-MnO_2_-PEG/C&D) for in vitro combination treatment. 4T1 cells incubated with H-MnO_2_-PEG/C&D for different periods of time were then imaged by a confocal fluorescence microscope (Fig. 3d). Both DOX and Ce6 fluorescence inside cells significantly enhanced with prolonging of incubation time. Interestingly, although Ce6 fluorescence mostly maintained in the cells cytoplasm after incubation for 12 h, obvious accumulation of DOX inside cell nuclei was found over time, indicating the gradual intracellular DOX release from H-MnO_2_-PEG/C&D after the break-up of H-MnO_2_ nanocarriers within acidic lysosomes after cellular uptake of those nanoparticles.

The combined PDT and chemotherapy based on H-MnO_2_-PEG/C&D was then demonstrated by treating 4T1 cells with H-MnO_2_-PEG/C plus light irradiation, H-MnO_2_-PEG/C&D in dark, or H-MnO_2_-PEG/C&D plus light. Cells for PDT treatment were exposed to the 660-nm light for 30 min (5 mW cm^−2^) after they were incubated with nanoparticles for 2 h. The cell viabilities were determined by the MTT assay after incubation for another 24 h (Fig. 3e). Compared with PDT alone (H-MnO_2_-PEG/C plus light) or chemotherapy alone (H-MnO_2_-PEG/C&D in dark), the combination therapy (H-MnO_2_-PEG/C&D plus light) was found to be the most effective in killing cancer cells by a synergistic manner under different drug concentrations.

### In vivo and ex vivo imaging with H-MnO_2_-PEG/C&D

**Fig. 4:**
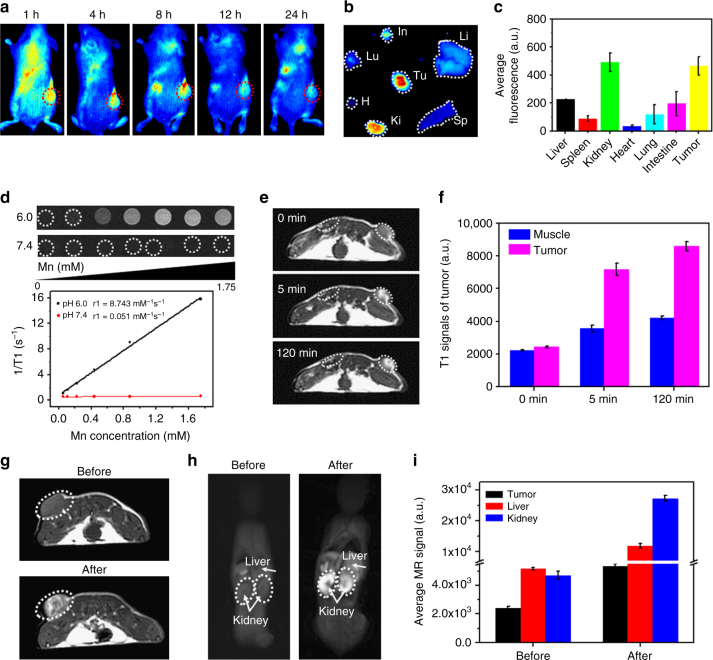
In vivo and ex vivo imaging with H-MnO_2_-PEG/C&D. **a** In vivo fluorescence images of 4T1 tumor-bearing mice taken at different time points post i.v. injection of H-MnO_2_-PEG/C&D (three mice per group). **b** Ex vivo fluorescence images of major organs and tumor dissected from mice at 24 h p.i. Sp, Ki, H, Lu, In, Li, and Tu stood for spleen, kidney, heart, lung, intestine, liver, and tumor, respectively. **c** Semi-quantitative analysis of ex vivo fluorescence images in different organs in (**b**). **d** T1-weighted MR images of the H-MnO_2_-PEG/C&D recorded using 3T MR scanner at different pH values (6 and 7.4). The transverse relativities (r1) were 8.743 and 0.051 mM^−1 ^s^−1^ for H-MnO_2_-PEG/C&D at pH 6 and 7.4, respectively. **e** T1-MR images of 4T1 tumor-bearing mice before and after local injection of H-MnO_2_-PEG/C&D within normal and tumor subcutaneous tissues (three mice per group). **f** Quantified T1-MR signals in muscle and tumor before and after injection of H-MnO_2_-PEG/C&D based on images in (**e**). **g**, **h** In vivo T1-MR images of a mouse taken before and 24 h post i.v. injection of H-MnO_2_-PEG/C&D shown in cross section (**g**) and longitudinal section (**h**). **i** Quantification analysis of T1-MR signals in liver, kidney, and tumor, before and 24 h post i.v. injection or H-MnO_2_-PEG/C&D nanoparticles. Date are presented as means ± s.d. (*n* = 3 mice per group)

After demonstrating the combination therapy function of H-MnO_2_-PEG/C&D in our in vitro experiments, we thus would like to test H-MnO_2_-PEG/C&D in the animal tumor model. We firstly used in vivo fluorescence imaging to track those nanoparticles in 4T1 tumor-bearing Balb/c mice after intravenous (i.v.) injection of H-MnO_2_-PEG/C&D (dose of MnO_2_ = 10 mg kg^−1^, Ce6 = 4.7 mg kg^−1^, and DOX = 4.5 mg kg^−1^) (Fig. 4a). The Ce6 fluorescence signals in the tumor region increased and reached a peak level at 8 h post injection, indicating the efficient tumor accumulation of those H-MnO_2_-PEG/C&D. Semi-quantitative biodistribution based on ex vivo imaging of major organs and tumor collected from 24 h post injection indicated the high-tumor uptake of H-MnO_2_-PEG/C&D (Fig. 4b, c). Notably, strong fluorescence of Ce6 found in kidneys of mice after injection of H-MnO_2_-PEG/C&D illustrated rapid renal clearance of Ce6 after decomposition of those nanoshells.

It is known that MnO_2_ nanoparticles were stable under neutral and basic pH, but would be decomposed into Mn^2+^ and O_2_ under acidic environment. As Mn^2+^ with five unpaired 3*d* electrons is known to be a T1-shortening agent in MR imaging^42^, MR imaging of H-MnO_2_-PEG/C&D incubated in buffer solutions with different pHs (6 and 7.4) for 6 h was conducted. The significant concentration-dependent brightening effect of H-MnO_2_-PEG/C&D samples were found in T1-MR images at pH 6, whereas the signals of nanoparticles in the neutral buffer solution appeared to be much weaker (Fig. 4d). Importantly, the r1 value (in terms of the molar concentration of Mn) was remarkably enhanced from the initial value of 0.051 mM^−1 ^s^−1^ at pH 7.4 to 8.743 mM^−1 ^s^−1^ after incubation in pH 6 buffer for 6 h, owing the decomposition of MnO_2_ nanoshells into paramagnetic Mn^2+^. To demonstrate the use of such MnO_2_ nanoshells for tumor-specific imaging, H-MnO_2_-PEG/C&D was directly injected into the tumor and the muscle on the opposite side for MR imaging (Fig. 4e). Interestingly, because of the acidic TME^29^, the tumor area exhibited significantly enhanced T1-MR contrast after injection of H-MnO_2_-PEG/C&D, whereas the muscle area with the same amount of nanoparticles injected showed much less T1 signal enhancement (Fig. 4f). This phenomenon gives the direct evidence that H-MnO_2_ with ultrasensitive pH-responsive T1-MR contrasting performance is particularly useful for tumor-specific imaging.

MR imaging was then conducted to image 4T1 tumor-bearing mice after i.v. injection of H-MnO_2_-PEG/C&D (Fig. 4g). At 24 h post injection of nanoparticles, the T1-MR signals showed twofold-positive enhancement in the tumor (Fig. 4i), demonstrating high-tumor accumulation of H-MnO_2_-PEG/C&D, consistent to the in vivo fluorescence imaging results. Moreover, strong T1 signals were also found in kidneys of those mice (Fig. 4h, i), suggesting fast renal clearance of Mn^2+^ ions decomposed from H-MnO_2_-PEG/C&D. Therefore, both fluorescence and MR imaging reveal that our PEGylated hollow MnO_2_ nanoshells with drug loading on one hand exhibit efficient passive tumor homing after systemic administration via the enhanced permeability and retention (EPR) effect, on the hand other could be gradually decomposed into free ions and molecules with rapid renal excretion.

### In vivo combined chemo-PDT treatment with H-MnO_2_-PEG/C&D

**Fig. 5:**
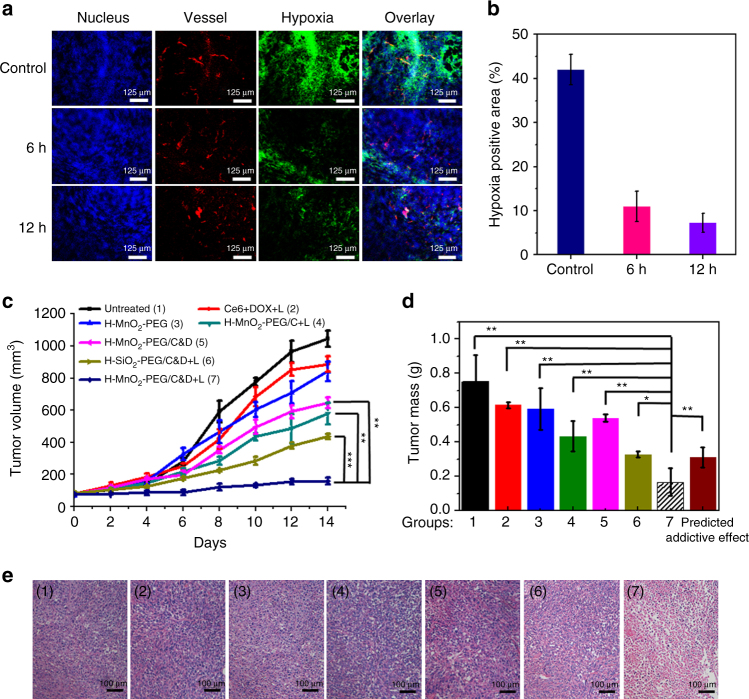
In vivo combined chemo-PDT treatment with H-MnO_2_-PEG/C&D. **a** Representative immunofluorescence images of 4T1 tumor slices collected from untreated control mice and mice 6 h and 12 h post i.v. injection with H-MnO_2_-PEG/C&D. The nuclei, blood vessels, and hypoxic areas were stained with DAPI (blue), anti-CD31 antibody (red), and anti-pimonidazole antibody (green), respectively (three mice per group). **b** Quantification of hypoxia areas in tumors at different time points post injection of our nanoparticles. **c** Tumor growth curves of different groups of mice after various treatments indicated. Error bars were based on standard errors of the mean (SEM) (six mice per group). **d** Average weight of tumors collected from mice at day 14th post initiation of various treatments. The predicted addictive effect was calculated by multiplying the tumor growth inhibition ratios of group 4 (PDT alone) and group 5 (chemotherapy alone). **e** H&E-stained tumor slices collected from mice post various treatments indicated. *p* values in (**c**) and (**d**) were calculated by Tukey’s post-test (***p < 0.001, **p < 0.01, or *p < 0.05)

It is known that cancer cells inside tumors are able to constitutively produce H_2_O_2_, whose level has been reported to be in the range of 10–100 μM in many types of solid tumors^41^. H-MnO_2_-PEG/C&D nanoparticles thus might be able to trigger the decomposition of H_2_O_2_ generated by cancer cells, producing O_2_ in situ to relieve tumor hypoxia. Therefore, a hypoxyprobe (pimonidazole) immunofluorescence assay was conducted to examine tumor slices extracted at different time points post i.v. injection of H-MnO_2_-PEG/C&D. The cell nuclei, blood vessels, and hypoxic areas were stained with 2-(4-amidinophenyl)-6-indolecarbamidine dihydrochloride (DAPI, blue), anti-CD31 antibody (red), and anti-pimonidazole antibody (green), respectively. Compared with the control group, tumor slices from mice treated with H-MnO_2_-PEG/C&D collected at different time points showed obviously reduced green fluorescence, indicating that the decomposition of H_2_O_2_ into O_2_ triggered by MnO_2_ accumulated in the tumor was able to greatly reduce the tumor hypoxia (Fig. 5a). Semi-quantitative analysis of hypoxia positive areas recorded from more than 15 confocal images per group further evidenced that i.v. injected H-MnO_2_-PEG/C&D could significantly reduce tumor hypoxia (Fig. 5b). Furthermore, it was found that H-MnO_2_-PEG without drug loading also could markedly decrease the tumor hypoxia signals (Supplementary Fig. 6). In contrast, for mice treated with H-SiO_2_-PEG, large hypoxia areas remained in their tumors (Supplementary Fig. 6).

Next, the efficacy of H-MnO_2_-PEG/C&D for enhanced PDT and chemotherapy was studied with the 4T1 mouse tumor model. As an inert control to MnO_2_ carriers, hollow mesoporous silica (H-SiO_2_) nanoshells were used to replace MnO_2_. Such H-SiO_2_ nanoshells with similar sizes to H-MnO_2_ after PEGylation also showed efficient co-loading of Ce6 and DOX, but no catalytic activity to H_2_O_2_ (Supplementary Fig. 7). In our in vivo treatment experiments, Balb/c mice bearing 4T1 tumors were divided into seven groups: untreated (group 1), free Ce6 + DOX with 660-nm light irradiation (group 2, Ce6 + DOX + L), H-MnO_2_-PEG (group 3), H-MnO_2_-PEG/C with 660-nm light irradiation (group 4, H-MnO_2_-PEG/C + L), H-MnO_2_-PEG/C&D (group 5), H-SiO_2_-PEG/C&D with 660-nm light irradiation (group 6, H-SiO_2_-PEG/C&D + L), and H-MnO_2_-PEG/C&D with 660-nm light irradiation (Group 7, H-MnO_2_-PEG/C&D + L). At 12 h post i.v. injection of those therapeutic agents (dose of MnO_2_ = 10 mg kg^−1^, SiO_2_ = 25 mg kg^−1^, Ce6 = 4.7 mg kg^−1^, and DOX = 4.5 mg kg^−1^), mice in group 2, 4, 6, and 7 were exposed to the 660-nm light for 1 h (5 mW cm^−2^).

The tumor sizes and mice body weights were measured in following 2 weeks (Fig. 5c; Supplementary Fig. 8). At day 14, the tumors of all mice were collected and weighted. Free drugs plus 660-nm laser irradiation (group 2) showed no appreciable effect on tumor growth, likely owing to the insufficient tumor retention of free Ce6 and DOX at such low doses. H-MnO_2_-PEG without laser irradiation showed no obvious suppressive effect on the tumor growth (group 3). For tumors on mice treated by H-MnO_2_-PEG/C exposed to 660-nm light irradiation (group 4, PDT alone), or H-MnO_2_-PEG/C&D without light irradiation (group 5, chemotherapy only), their growth was partially delayed. Combination therapy with H-SiO_2_-PEG/C&D plus light irradiation offered more significant tumor growth-inhibition effect (group 6). Notably, in the combination treatment group of H-MnO_2_-PEG/C&D with 660-nm light, the tumors showed the slowest growth speed and smallest volumes at the end of treatment. Importantly, the therapeutic efficacy of our combination therapy with H-MnO_2_-PEG/C&D was obviously stronger than the predicted addictive effect (Fig. 5d), indicating the significant synergistic effect by the combined PDT and chemotherapy delivered by H-MnO_2_-PEG/C&D. Furthermore, hematoxylin and eosin (H&E) staining of tumor slices also showed that the majority of tumor cells were severely damaged in the group of H-MnO_2_-PEG/C&D with 660-nm light (Fig. 5e). After 14 days, H&E-stained images of major organs from the combination therapy group suggested that our H-MnO_2_-PEG/C&D induced no obvious toxic side effects to mice (Supplementary Fig. 9).

### Immunological responses after the combined chemo-PDT

**Fig. 6:**
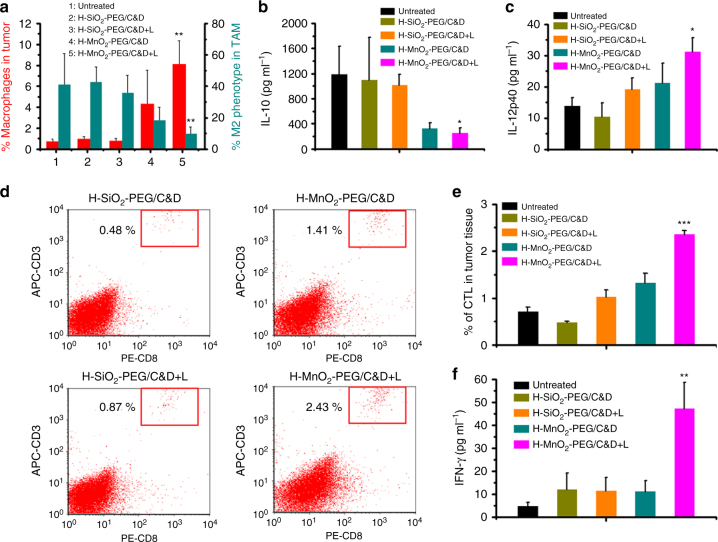
Immunological responses after combined chemo-PDT with H-MnO_2_-PEG/C&D. **a** Macrophages infiltration and polarization within tumors post various treatments. CD11b^+^CD206^+^ cells were defined as M2 phenotype macrophages (six mice per group). The total number of macrophages infiltrated in tumors markedly increased post the combined chemo-PDT, which also resulted in polarization of M2 phenotype TAM towards M1 TAM. **b**, **c** The levels of IL-10 (**b**) and IL-12p40 (**c**) in the supernatant of tumors post various treatments. **d**, **e** Representative flow cytometry data of cytotoxic T lymphocytes (CTL) infiltration in tumors. CD3^+^CD8^+^ cells were defined as CTLs. **f** The production of IFN-γ in sera of mice post various treatments. *p* values were calculated by Tukey’s post-test between group 5 (H-MnO_2_-PEG/C&D + L) and group 1 (untreated) (***p < 0.001, **p < 0.01, or *p < 0.05) (compared with untreated). Date are presented as means ± s.d. (*n* = 6)

In recent years, extensive evidences have highlighted the pivotal roles of TME in effective cancer immunotherapy^43^. For instance, M2 type TAM and regulatory T cells (Treg) are immunosuppressive cells that can promote tumor progression via inhibiting anti-tumor immunities^44, 45^. It has been uncovered that the hypoxic TME could promote the recruitment of Treg into tumors^12^ and tune TAM into M2-like phenotype cells^12, 13, 46^. Considering the ability of our nanoparticles to relieve tumor hypoxia, we thus worked whether combination treatment with such H-MnO_2_-PEG/C&D would have any effect to the tumor immunology. The changes of TAM and Treg at day 5 post treatments in tumors were firstly examined using flow cytometry assay. Interestingly, compared with the untreated group, tumors on mice with i.v. injection of H-MnO_2_-PEG/C&D after light exposure (combined chemo-PDT) showed significantly enhanced macrophages infiltration within tumor from 0.8% to 8%, together with largely reduced population of M2 phenotype TAMs from 40% to 9.5% among total TAM (Fig. 6a). Consistent to a previous report^47^, injection of MnO_2_ by itself could also induce a certain level of TAM polarization, although to be much less extend as found in our work. In contrast, no significant change of TAM phenotypes was observed in tumor after combination treatment with H-SiO_2_-based nano-platform. Meanwhile, the secretion of IL-10 (predominant cytokine secreted by M2 macrophages) in the supernatant of tumor lysates also significantly decreased by 3.77 times for the H-MnO_2_-PEG/C&D-injected mice plus light irradiation (Fig. 6b), whereas the secretion of IL-12 (predominant cytokine secreted by M1 macrophages) in tumor showing significant upregulation (Fig. 6c), both indicating significant M2 to M1 polarization for TAM within tumors post chemo-PDT with H-MnO_2_-PEG/C&D.

In the meanwhile, we also checked the populations of different sub-groups of T cells in tumors post various types of treatment by flow cytometry. It was found that there were more cytotoxic T lymphocytes (CTL) infiltrated in the tumor after treatment with H-MnO_2_-PEG/C&D plus light irradiation (Fig. 6d, e). The high expression of interferon gamma (IFN-γ) in the serum confirmed the CTL-mediated cellular immunity induced by such treatment (Fig. 6f)^48^. Moreover, it was also observed that such treatment with H-MnO_2_-PEG/C&D could slightly reduce the population of immunosuppressive Treg within tumors (Supplementary Fig. 10). In contrast, treatment with H-SiO_2_-PEG/C&D (plus light irradiation) without the hypoxia modulating ability exerted no obvious effect to the sub-populations of T cells. These distinguished effects may be attributed to the TME modulation capacity of H-MnO_2_-based nano-platform and the subsequently enhanced chemo-PDT treatment to induce the release of tumor-associated antigens^49, 50^, which would activate dendritic cells and then the recruitment of effector T cells into tumors. Furthermore, extensive evidences^51^ have demonstrated that PDT treatment could induce the release of heat shock proteins to facilitate the antigen cross-presentation and finally activate CTL-mediated cellular immunity (e.g., IFN-γ expression). In other words, besides killing tumor cells, the combined chemo-PDT treatment with our H-MnO_2_-PEG/C&D nano-platform could simultaneously induce CTL-mediated anti-tumor immunities and moderate the immunosuppressive microenvironment inside tumors, favorable for immune killing of tumor cells, which have survived after the first round of treatment by chemo-PDT.

### H-MnO_2_-PEG/C&D plus anti-PD-L1 checkpoint blockade

**Fig. 7:**
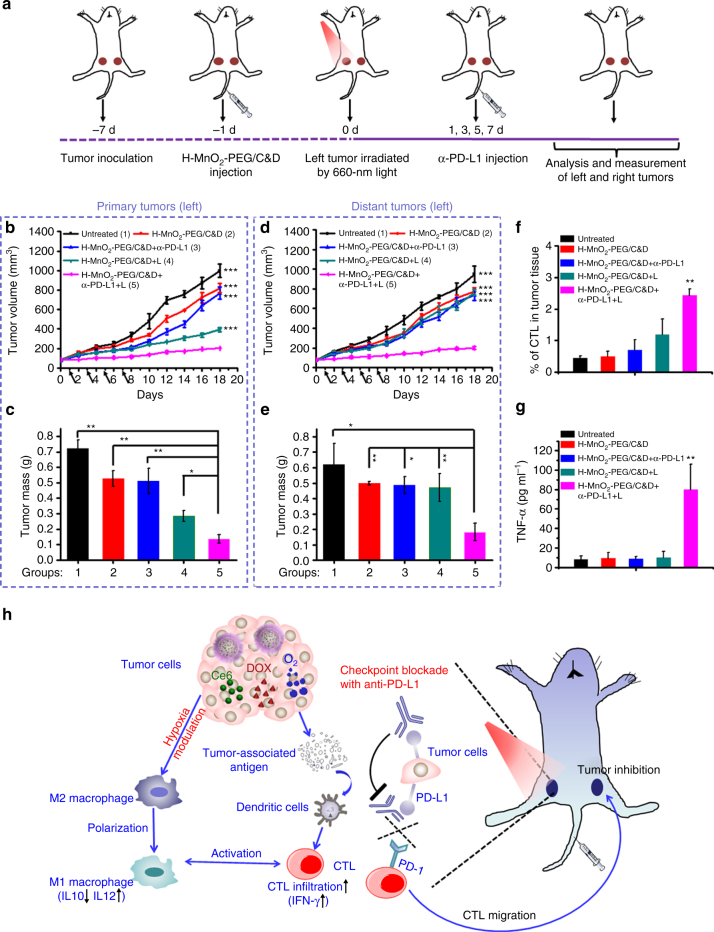
The abscopal effect of H-MnO_2_-PEG/C&D in combination with anti-PD-L1 (α-PD-L1) checkpoint blockade. **a** Schematic illustration of H-MnO_2_-PEG/C&D and anti-PD-L1 combination therapy. **b**, **d** Primary (**b**) and distant (**d**) tumors growth curves of different groups of mice after various treatments indicated. Error bars are based on SEM (six mice per group). The arrows represent the time points of anti-PD-L1 administration. **c**, **e** Average weights of primary (**c**) and distant (**e**) tumors collected from mice 18 days after initiation of various treatments. **f** CTL infiltration in distant tumors. CD3^+^CD8^+^ cells were defined as CTLs. **g** The production of TNF-α in sera of mice determined on the 9th day post various treatments. **h** The proposed mechanism of anti-tumor immune responses induced by H-MnO_2_-PEG/C&D in combination with anti-PD-L1 therapy. *p* values were calculated by Tukey’s post-test (***p < 0.001, **p < 0.01, or *p < 0.05)

It is well known that tumor cells themselves could induce the CTL exhaustion through PD-L1 signaling pathway for immune evasion^45^. Recently, PD-1/PD-L1 checkpoint-blockade strategies approved by the U.S. Food and Drug Administration (FDA) to promote anti-tumor immunities by inhibiting CTL exhaustion has demonstrated to be a promising cancer immunotherapy method with exciting clinical results in cancer treatment^52^. Encouraged by the robust cellular immunity induced by the combined chemo-PDT with H-MnO_2_-PEG/C&D, we hypothesized that such a novel TME-responsive/modulating treatment strategy might have a synergistic effect with PD-L1 checkpoint blockade. In the following study, a bilateral breast tumor model was developed by subcutaneously injecting 4T1 cells into both the left and right flank regions of mice (Fig. 7a). The left and right tumors were designated as the primary and distant tumors, respectively. When those tumors reached 80 mm^3^, mice were randomly divided into five groups (*n* = 6 per group): (1) PBS (untreated); (2) H-MnO_2_-PEG/C&D injection; (3) H-MnO_2_-PEG/C&D injection plus anti-PD-L1 treatment; (4) H-MnO_2_-PEG/C&D injection followed by light irradiation (H-MnO_2_-PEG/C&D + L); (5) “H-MnO_2_-PEG/C&D + L” treatment together with anti-PD-L1 treatment. Light irradiation for group 4 and 5 was conducted only on the left tumors (primary tumors) using the 660-nm LED light for 1 h (5 mW cm^−2^), whereas the right distant tumors were spared from light-induced PDT. PD-L1 blockade therapy was adopted at day 1, 3, 5, and 7 post chemo-PDT treatment by i.v. injection of anti-PD-L1 (dose = 750 μg kg^−1^) (Fig. 7a).

As expected, H-MnO_2_-PEG/C&D-based chemo-PDT treatment showed further improved therapeutic efficacy in combating the primary tumor progression (the left tumor with light exposure) when combining with PD-L1 blockade, in comparison to chemo-PDT alone with H-MnO_2_-PEG/C&D (Fig. 7b, c). Most notably, for tumors on the right side (distant tumors) without light exposure, the chemo-PDT with H-MnO_2_-PEG/C&D on primary tumors together with PD-L1 blockade could also effectively delay their growth, whereas the growth of distant tumors in all other control groups were not significantly affected (Fig. 7d, e). Therefore, such chemo-PDT treatment with H-MnO_2_-PEG/C&D in combination with PD-L1 blockade could not only effectively kill primary tumor cells with direct light exposure, but also inhibit the growth of distant tumors (e.g., tumors located deeply inside the body, or too small to be detected before treatment) spared from light exposure.

To understand such phenomenon, CTL infiltration in distant tumors without PDT treatment was then examined. Interestingly, the CTL recruitment within the distant tumor for the combination treatment group (group 5) increased remarkably. In marked contrast, the CTL infiltration levels within distant tumors in other control groups, including chemo-PDT with H-MnO_2_-PEG/C&D (no PD-L1 blockade) and H-MnO_2_-PEG/C&D injection plus anti-PD-L1 (no light irradiation on the primary tumor), were not significantly affected (Fig. 7f; Supplementary Fig. 11). Furthermore, we also checked the level of TNF-α, an important indicator of anti-tumor systemic immune responses, in sera of mice at day 9th post the irradiation of primary tumors. It was shown that the chemo-PDT with H-MnO_2_-PEG/C&D together with PD-L1 blockade could induce the highest level of TNF-α secretion in serum in comparison to all other control groups (Fig. 7g). All these interesting results indicate that although TME-modulating treatment with H-MnO_2_-PEG/C&D could reverse the immunosuppressive microenvironment inside tumors and trigger CTL-mediated anti-tumor immunities, the PD-L1 blockade could further potentiate the generation of tumor antigen-specific CTL effector cells, which are then migrated into the distant tumors to kill tumor cells there.

Subsequently, we also conducted T cell blocking experiments to confirm the involvement of T cells in the efficient abscopal response. 4T1 tumor-bearing mice were treated with H-MnO_2_-PEG/C&D + anti-PD-L1 + light irradiation as described before, and then received i.p. injection of anti-CD4, anti-CD8, or mouse IgG (as the control) at a dose of 200 μg per mouse on day 0 and 5. As expected, mouse IgG injection did not affect the therapeutic outcomes of our combination treatment in inhibiting both primary and distant tumors. In marked contrast, blocking of either CD4^+^ T cells or CD8^+^ T cells greatly impaired the capability of H-MnO_2_-PEG/C&D (with light) + anti-PD-L1 treatment in inhibiting both primary and distant tumors, particularly at later time points post treatment (Supplementary Fig. 12). Our results indicate that both CD4^+^ and CD8^+^ T cells are essential not only to the abscopal effect in inhibiting distant tumors, but also important to the growth inhibition of primary tumors after the combined chemo-PDT-immunotherapy.

## Discussion

As depicted in Fig. 7h, our developed novel H-MnO_2_-PEG/C&D nano-platform with efficient tumor-homing capacity could effectively release the chemotherapeutic drug and photodynamic agent upon responsive to tumor acidic microenvironment. Meanwhile, this novel nano-platform could also relieve the hypoxic condition via inducing decomposition of endogenous H_2_O_2_ inside the tumor to further promote photodynamic cancer cell killing. More significantly, the combined chemo-PDT with H-MnO_2_-PEG/C&D would result in a number of immunological consequences: (1) The TME modulation capacity of H-MnO_2_-PEG-based nano-platform could effectively shape the immunosuppressive microenvironment to favor anti-tumor immunities (e.g., TAM polarization from M2 to M1). (2) Besides eliminating primary tumor cells, the enhanced chemo-PDT treatment may generate tumor-associated antigens released from the apoptotic or necrotic tumor cells^50^, which would be engulfed by antigen presenting cells like dendritic cells to induce anti-tumor cellular immunities (e.g., CTL infiltration in tumors). (3) Furthermore, the above mentioned effects would greatly facilitate the synergistic effect between chemo-PDT treatment and PD-L1 checkpoint blockade. In such comprehensive treatment strategy, the tumor-killing CTL effector cells generated after the chemo-PDT treatment plus PD-L1 blockade would migrate into other distant tumors and destruct those tumor cells expressing the same type of tumor-associated antigens, promising for effectively killing of tumor cells that cannot be directly irradiated by light during PDT, as well as for tumor metastasis inhibition.

In summary, we have fabricated hollow mesoporous MnO_2_ nanoshells with PEG coating and dual-drug loading (H-MnO_2_-PEG/C&D) as a multifunctional theranostic platform that is responsive to TME and be able to modulate TME, for enhanced cancer combination chemo-PDT therapy, which further favors anti-tumor immunities to promote cancer immunotherapy. Compared with previously reported MnO_2_ nanostructures, hollow mesoporous H-MnO_2_ nanoshells developed here show advantages in highly effective drug loading as well as precisely controlled drug release. The ultrasensitive pH responsiveness of H-MnO_2_-PEG/C&D enables tumor-specific MR imaging as well as efficient drug release under acidic TME pH. The relieved tumor hypoxia by MnO_2_-triggered decomposition of endogenous H_2_O_2_ inside tumors offered remarkable benefits not only for improving the efficacy of chemo-PDT, but also for reversing the immunosuppressive TME to favor anti-tumor immunities post treatment. Further combination of H-MnO_2_-PEG/C&D-based chemo-PDT with PD-L1 checkpoint blockade offers an abscopal effect to inhibit the growth of not only primary tumors but also distant tumors without light exposure likely through the CTL migration. Subsequent T-cells depletion experiments demonstrate that this combinational chemo-PDT-immunotherapy strategy would inhibit tumor growth mainly through modulating T cells-mediated immunities. With inherent biodegradability, our H-MnO_2_-based theranostic nano-platform may indeed find significant potential in clinical translation to allow the combination of chemotherapy, PDT, and cancer immunotherapy, which acting together after modulation of TME could offer a synergistic comprehensive effect in battling cancer.

## Methods

### Materials

Treaethyl orthosilicate (TEOS), poly(allylamine hydrochloride) (PAH, MW ≈ 15,000), and polyacrylic acid (PAA, MW ≈ 1800) were purchased from Sigma-Aldrich. Potassium permanganate (KMnO_4_) and sodium carbonate (Na_2_CO_3_) were obtained from Sinopharm Chemical Reagent CO., Ltd. (China). Hydrogen peroxide (H_2_O_2_) 30 wt% solution and Chlorin e6 (Ce6) were purchased from J&K chemical CO. SOSG reagent was purchased from Molecular Probes (Eugenr, OR). DOX was purchased from Beijing HuaFeng United Technology Co. Ltd. PEG polymers were obtained from JiaXingBoMei, China.

### Synthesis of H-MnO_2_-PEG/C&D

Solid silica nanoparticles (sSiO_2_) were synthesized following the reported method^53^. Then an aqueous solution of KMnO_4_ (300 mg) was dropwise added into the suspension of sSiO_2_ (40 mg) under ultrasonication. After 6 h, the precipitate was obtained by centrifugation at 14,800 rpm. The as-prepared mesoporous MnO_2_-coated sSiO_2_ was dissolved in 2 M Na_2_CO_3_ aqueous solution at 60 °C for 12 h. The obtained hollow mesoporous MnO_2_ nanoshells (H-MnO_2_) were centrifuged and washed with water several times. Overall, 5 mL H-MnO_2_ solution (2 mg mL^−1^) was then added to 10 mL PAH solution (5 mg mL^−1^) under ultrasonication. After stirring for 2 h, the above solution was centrifuged and washed with water. The obtained H-MnO_2_ /PAH solution was dropwisely added into 10 mL PAA (5 mg mL^−1^) under ultrasonication. After 2 h of stirring, the above solution was centrifuged and washed with water, before it was mixed with 50 mg mPEG-5K-NH_2_ under ultrasonication for 30 min. After adding 15 mg EDC and stirring for 12 h, the prepared H-MnO_2_-PEG was collected by centrifugation and washed with water for three times. For Ce6 and DOX loading, the H-MnO_2_-PEG solution (0.2 mg mL^−1^) was mixed with different concentrations of Ce6 and DOX for 12 h. Ce6 and DOX were co-loaded into H-MnO_2_-PEG with appropriate concentrations, yielding H-MnO_2_-PEG/C&D which was used for further experiments.

### Characterizations

Transmission electron microscopy (TEM, FEI Tecnai F20, acceleration voltage = 200 KV) was applied to characterize the morphology of nanoparticles. UV–vis spectra were measured with a PerkinElmer Lambda 750 UV–vis-NIR spectrophotometer. The sizes and zeta potentials of nanoparticles were determined by a Malvern zetasizer (ZEN3690, Malvern, UK). Surface area and pore size were measured by Surface Area and Porosity Analyzer (Micromeritics Instrument Corp. ASAP2050). The dissolved O_2_ was measured with an oxygen probe (JPBJ-608 portable Dissolved Oxygen Meters, Shanghai REX Instrument Factory).

### Degradation and drug release studies

H-MnO_2_-PEG was incubated with different pH values of PBS (5.5, 6.5, and 7.4) for different durations. At the given time points, the solution was measured by TEM and UV–vis spectrometer for characterizations. To study the Ce6 and DOX release, a solution of H-MnO_2_-PEG/C&D was dialyzed against PBS with different pH values (5.5, 6.5, and 7.4) under room temperature. The amounts of Ce6 and DOX release at different time points were measured by UV–vis spectra.

### Detection of SO

SOSG, which is super-sensitive to produced SO, can be employed for SO detection^54^. Free Ce6 and H-MnO_2_-PEG/C with or without H_2_O_2_ added were incubated with SOSG (2.5 μM), and then irradiated under 660-nm light with various periods of time in N_2_ atmosphere (5 mW cm^−2^). The generated SO was determined by the recovered SOSG fluorescence under 494-nm excitation.

### In vitro cell experiments

4T1 murine breast cancer cell line was originally obtained from American Type Culture Collection (ATCC) and then incubated under 37 °C within 5% CO_2_ atmosphere. For cell toxicity assay, cells were seeded into 96-well plates (1 × 10^4^ per well) until adherent and then incubated with series concentrations of H-MnO_2_-PEG. The standard thiazolyl tetrazolium (MTT, Sigma-Aldrich) test was applied to measure the cell viabilities relative to untreated cells.

For confocal fluorescence imaging, 4T1 cells were cultured in 24-well plates containing H-MnO_2_-PEG/C&D (Ce6 = 10 μM, DOX = 5.7 μg mL^−1^) in the dark for different incubation time (1, 4, 8, and 12 h). After washing with PBS for three times, the cells were labeled with 4′, 6-diamidino-2-phenylindole (DAPI) and imaged by a laser scanning confocal fluorescence microscope (Leica SP5).

For PDT, 4T1 cells were cultured in 96-well plates and incubated with H-MnO_2_-PEG/C or free Ce6 with various concentrations. The 96-well plates were moved to a transparent box ventilated with either oxygen or nitrogen in advance for 2 h. Afterwards, cells were exposed to the 660-nm light for 30 min (5 mW cm^−2^) within either oxygen or nitrogen atmosphere. Cells were then replaced with fresh media and incubated for another 24 h. The cells viabilities were determined by the MTT assay.

For in vitro combination therapy, 4T1 cells seeded in 96-well plates were incubated with different concentrations of H-MnO_2_-PEG/C or H-MnO_2_-PEG/C&D for 2 h and then treated with or without 660-m light irradiation (5 mW cm^−2^, 30 min). After incubation for another 2 h, the cells were transferred into fresh media and incubated for another 24 h before the MTT assay to measure relative cell viabilities.

### Animal models

Female Balb/c mice (6–8 weeks) were purchased from Nanjing Peng Sheng Biological Technology Co.Ltd, and then implemented in accordance with protocols approved by Soochow University Laboratory Animal Center. 4T1 cells (5 × 10^6^) suspended in 30 μL of PBS were subcutaneously injected into the back of mouse. The mice bearing 4T1 tumors were treated when the volume of tumor reached about ~60 mm^3^.

### In vivo imaging

In vivo fluorescence imaging was performed using the Maestro in vivo fluorescence imaging system (Cri inc.). MR imaging was conducted under a 3.0-T clinical MRI scanner (GE healthcare, USA) with a special coil for small animal imaging.

### Immunohistochemistry

4T1 tumor-bearing mice were i.v. injected with PBS or H-MnO_2_-PEG/C&D. At 6 or 12 h post injection, tumors were surgically excised 90 min after intraperitoneal injection with pimonidazole hydrochloride (60 mg kg^−1^) (Hypoxyprobe-1 plus kit, Hypoxyprobe Inc.), which was reductively activated in hypoxic cells and formed stable adducts with thiol groups in proteins. For immunofluorescence staining, OCT compound (Sakura Finetek) was employed to prepare frozen sections of the tumors. For detection of pimonidazole, the tumor sections were treated with mouse anti-pimonidazole primary antibody (dilution 1:200, Hypoxyprobe Inc.) and Alex 488-conjugated goat anti-mouse secondary antibody (dilution 1:200, Jackson Inc.) following the kit’s instructions. Tumor blood vessels were stained by rat anti-CD31 mouse monoclonal antibody (dilution 1:200, Biolegend) and Rhodamine-conjugated donkey anti-rat secondary antibody (dilution 1:200, Jackson), subsequently. Cell nuclei were stained with DAPI (dilution 1:5000, Invitrogen). The obtained slices were observed by a confocal microscopy (Leica SP5).

### In vivo cancer treatment

4T1 tumor-bearing mice were i.v. injected with 200 μL of PBS, free Ce6 + DOX, H-MnO_2_-PEG/C, H-SiO_2_-PEG-C&D, or H-MnO_2_-PEG/C&D (dose of MnO_2_ = 10 mg kg^−1^, SiO_2_ = 25 mg kg^−1^, Ce6 = 4.7 mg kg^−1^, and DOX = 4.5 mg kg^−1^), respectively. The 660-nm light irradiation was conducted at 12 h post injection (5 mW cm^−2^, 1 h). Tumor sizes and body weights were monitored every 2 days for 2 weeks. The tumor volume was calculated following the formula: volume = width^2^ × length/2. The tissue and tumor slices were stained by H&E following the standard protocol.

To evaluate the immunological effects of chemo-PDT combination treatment, 4T1 tumor-bearing mice were randomly divided into 5 groups and i.v. injected with 200 μL of PBS, H-SiO_2_-PEG/C&D (with or without laser irradiation), or H-MnO_2_-PEG/C&D (with or without laser irradiation, dose of MnO_2_ = 10 mg kg^−1^, SiO_2_ = 25 mg kg^−1^, Ce6 = 4.7 mg kg^−1^, and DOX = 4.5 mg kg^−1^). The 660-nm light irradiation was conducted at 12 h (5 mW cm^−2^, 1 h) post injection. At day 5 post irradiation, mice were sacrificed and tumors were collected for the immunological evaluations. Briefly, tumor tissues were cut into small pieces and put into a glass homogenizer containing PBS solution (pH = 7.4). Then, the single cell suspension was prepared by gentle pressure with the homogenizer without addition of digestive enzyme^55^. Finally, the supernatant of tumors were collected to determine IL-10 and IL-12p40 levels using ELISA assay (eBioscience). Meanwhile, cells were stained with fluorescence-labeled antibodies after the removal of red blood cells (RBC) using the RBC lysis buffer. For macrophage polarization, cells were stained with anti-CD206-FITC, anti-CD11b-PE and anti-F4/80-AlexaFluor 647 (eBioscience) antibodies according to the manufacturer’s protocols. CD11b^+^F4/80^+^ and CD11b^+^F4/80^+^CD206^+^ cells were defined as macrophages and M2 phenotype macrophages, respectively. For regulatory T cells (Treg) evaluation, cells were stained with anti-CD3-FITC, anti-FoxP3-PE, anti-CD4-PerCP, and anti-CD8-APC (eBioscience) antibodies according to the manufacturer’s protocols. Meanwhile, cells were stained with anti-CD3-APC (BD Biosciences) and anti-CD8-PE (BD Biosciences) for evaluating cytotoxic T lymphocytes (CTL) infiltration. CD3^+^CD4^+^FoxP3^+^ and CD3^+^CD8^+^ cells were defined as Treg and CTL, respectively. In addition, interferon gamma (IFN-γ) and tumor necrosis factor alpha (TNF-α) in the sera of mice were also determined using ELISA assay (eBioscience).

To develop the bilateral tumor model, 4T1 cells were subcutaneously injected into left (primary tumor) and right (distant tumor) flank. After one week, those mice were i.v. injected with 200 μL of PBS, H-MnO_2_-PEG/C&D (dose of MnO_2_ = 10 mg kg^−1^, Ce6 = 4.7 mg kg^−1^, and DOX = 4.5 mg kg^−1^), or H-MnO_2_-PEG/C&D + anti-PD-L1, respectively. Twelve hours after injection, the primary tumors were irradiated with a 660-nm light (5 mW cm^−2^, 1 h). Then, mice were i.v. injected with anti-PD-L1 antibody at a dose of 750 μg kg^−1^ at day 1, 3, 5, and 7 post irradiation. Primary and distant tumor sizes and body weights were monitored every 2 days. At day 9 post irradiation, the sera of mice were collected using orbital sinus blood sampling to determine TNF-α. Meanwhile, T cells infiltration within bilateral tumors were evaluated using flow cytometry assay.

The abscopal therapeutic effect of H-MnO_2_-PEG/C&D + anti-PD-L1 was evaluated on 4T1 tumor-bearing mice with CD4^+^ T cell or CD8^+^ T cell depletion. When the tumors reached ~100 mm^3^, mice were i.v. injected with H-MnO_2_-PEG/C&D (dose = 200 μg per mouse) and exposed to the 660-nm light (5 mW cm^−2^, 1 h) at 12 h post injection. Then, mice were i.v. injected with anti-PD-L1 antibody at a dose of 750 μg kg^−1^ at day 1, 3, 5, and 7 post irradiation. Anti-CD4 (for CD4^+^ T cell depletion, GK1.5, BioXcell), anti-CD8 (for CD8^+^ T cell depletion, 2.43, BioXcell) or mouse IgG (Control, SouthernBiotech) were intraperitoneally injected to the mice (200 μg per mouse) on day 0 and 5 post the treatment. Primary and distant tumor sizes and body weights were measured every 2 days. Tumor volumes were calculated as width^2^ × length/2

### Data availability

All other remaining data are available within the article and supplementary files, or available from the authors upon request.

## Electronic supplementary material

Supplementary Information
