# Supplementary material for: Hollow MnO2 as a tumor-microenvironment-responsive biodegradable nano-platform for combination therapy favoring antitumor immune responses
Source: Nat Commun. 2017 Oct 12;8:902. doi: 10.1038/s41467-017-01050-0 (PMC5638920; doi:10.1038/s41467-017-01050-0)
Supplement: Supplementary file 1 — Supplementary Information [file 41467_2017_1050_MOESM1_ESM.pdf]

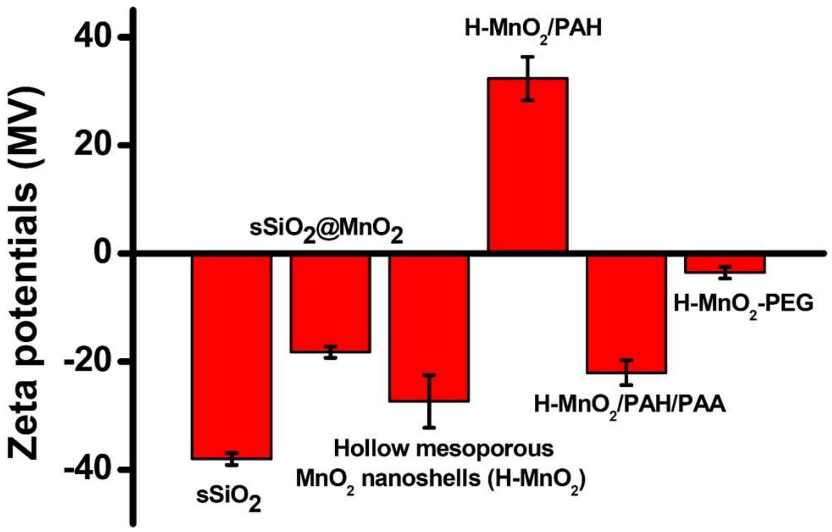

Supplementary Figure 1. The changes of zeta potentials for nanoparticles obtained at different steps of fabracation. Date are presented as means ± standard deviation (s.d.) (n = 3).

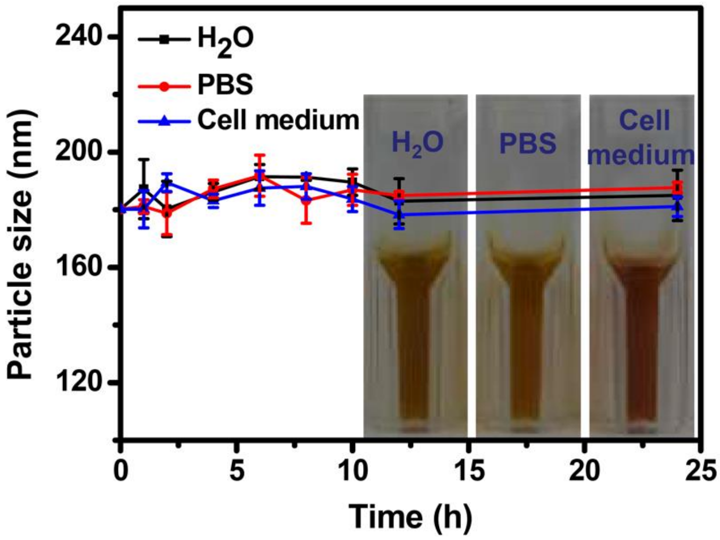

Supplementary Figure 2. DLS measured sizes of H-MnO_2-PEG in different solutions including water, phosphate buffered saline (PBS) and serum-containing cell medium. Date are presented as means ± s.d. (n = 3).

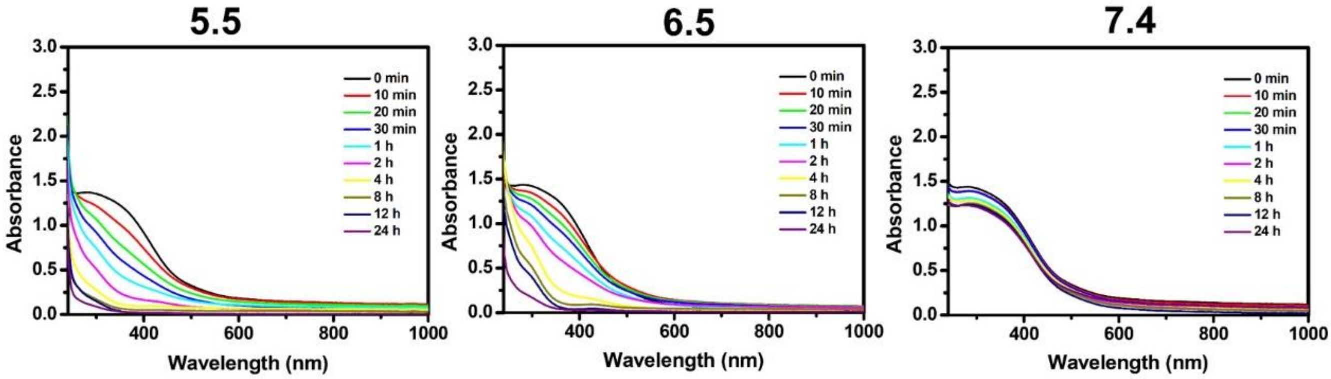

Supplementary Figure 3. The degradtion behavior of H-MnO_2-PEG/C&D incubated in PBS with various pH values (5.5, 6.5 and 7.4) measured by UV-vis spectra.

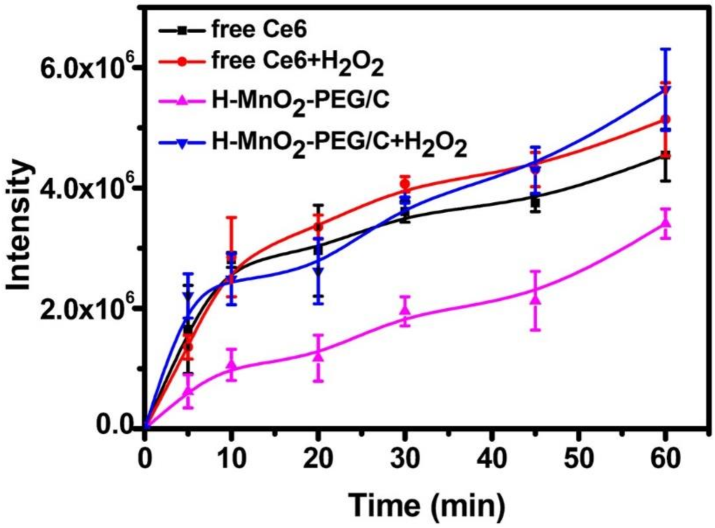

Supplementary Figure 4. The generation of singlet oxygen determined by the increased SOSG fluorescence for free Ce6 and H-MnO_2-PEG/C with or without addition of H_2O_2. Date are presented as means ± s.d. (n = 3).

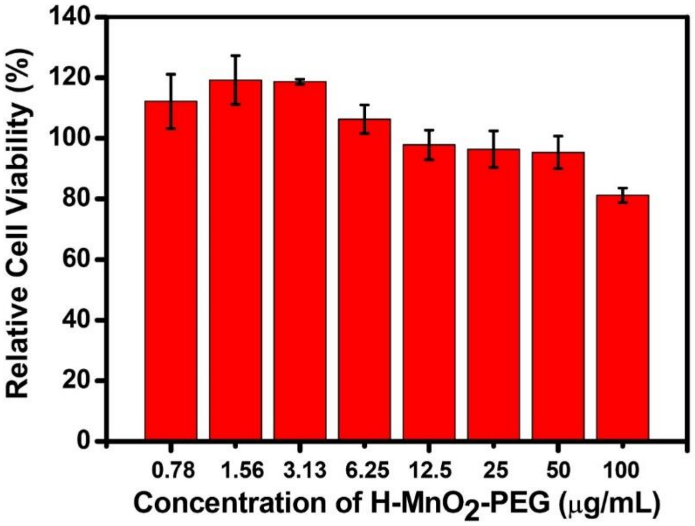

Supplementary Figure 5. Relative viabilities of 4T1 cells after being incubated with various concentrations of H-MnO_2-PEG in the dark for 24 h. Date are presented as means ± s.d. (n = 5).

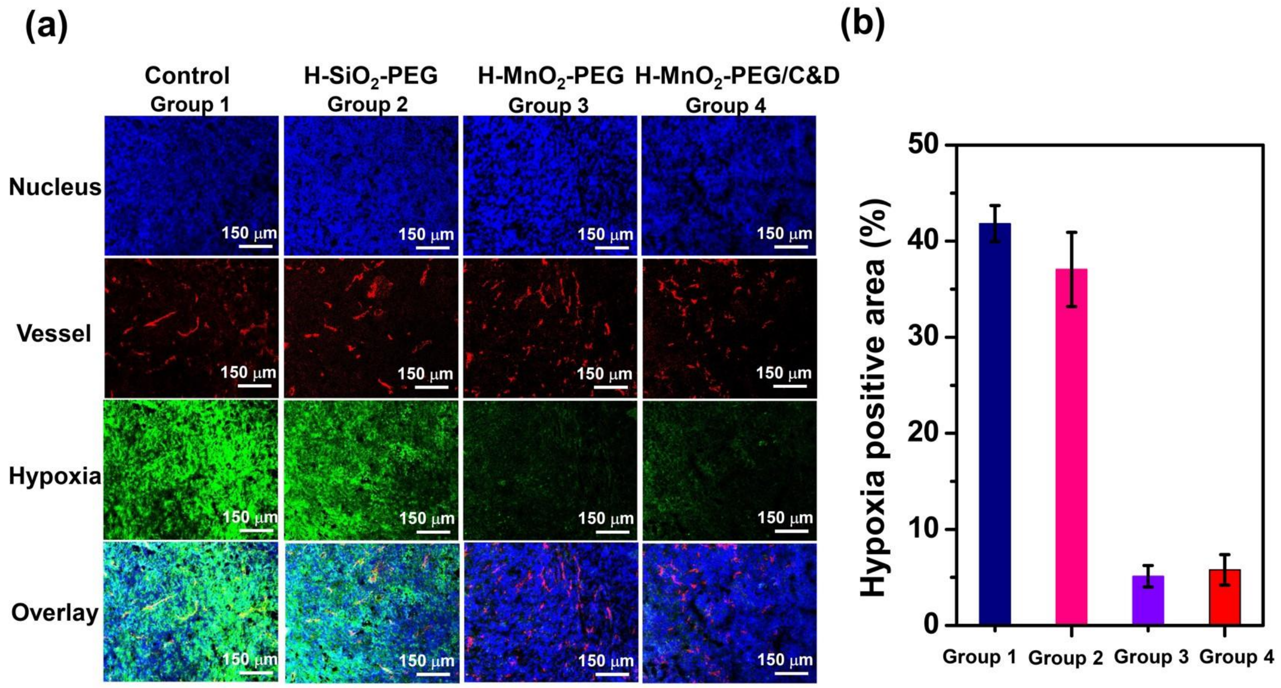

Supplementary Figure 6. (a) Representative immunofluorescence images of Hypoxyprobe-stained tumor slices collected at 12 h post i.v. injection with different agents. The nuclei, blood vessels and hypoxia areas were stained with DAPI (blue), anti-CD31 antibody (red) and anti-pimonidazole antibody (green), respectively (3 mice per group). (b) Quantification of tumor hypoxia signals for different groups shown in (a). For each tumor, more than 10 micrographs were analyzed to obtain the average value. Date are presented as means ± s.d. (n = 3 mice per group).

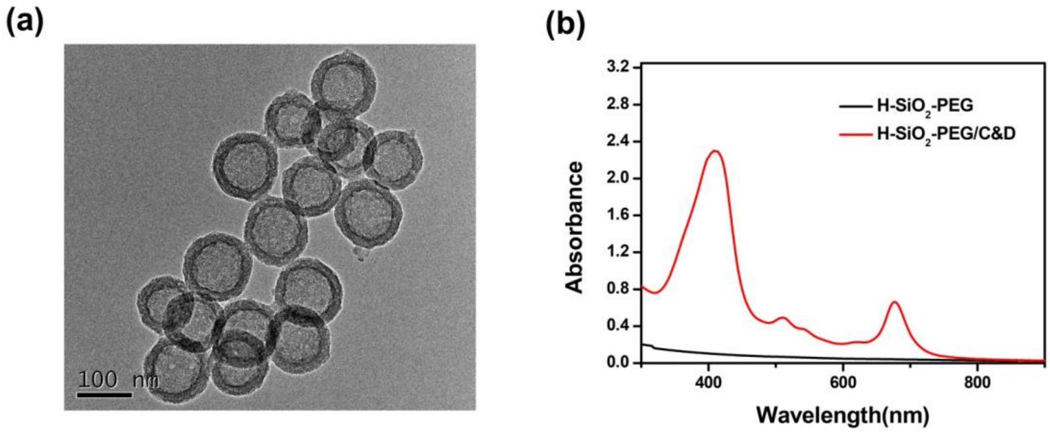

Supplementary Figure 7. (a) TEM imgae of H-SiO_2-PEG/C&D. (b) UV-vis spectra of H-SiO_2-PEG and H-SiO_2-PEG/C&D.

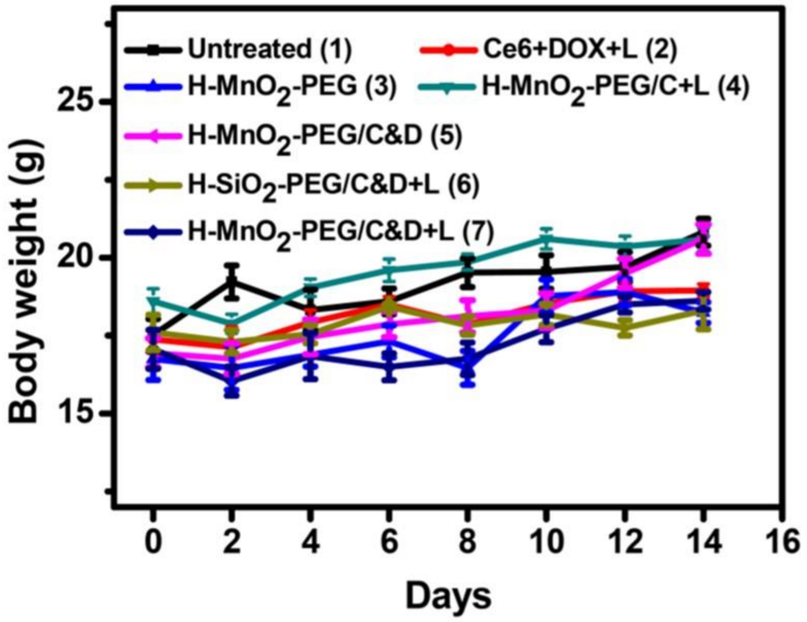

Supplementary Figure 8. Average body weights of mice after various treatments indicated. Error bars are based on the standard errors of the mean (n = 6).

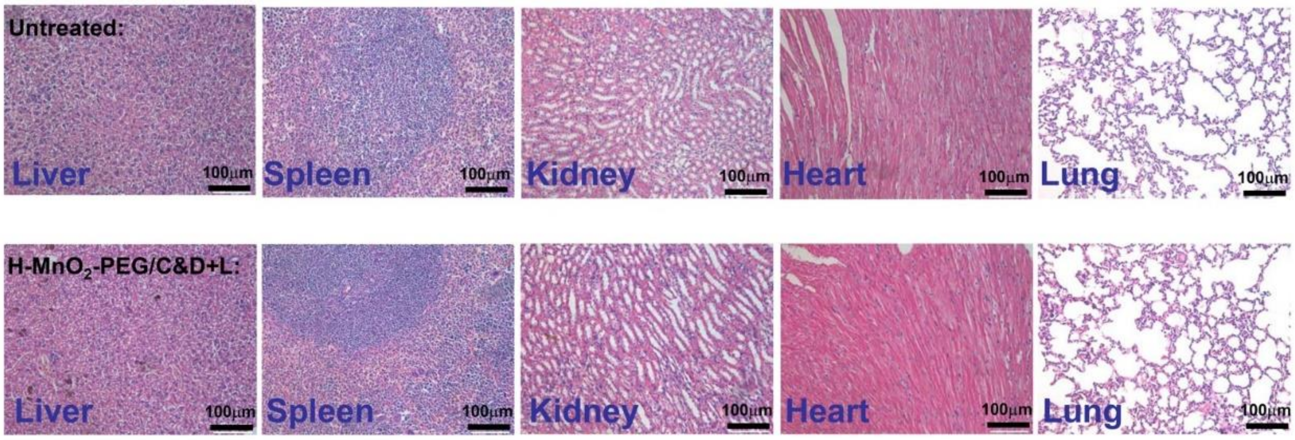

Supplementary Figure 9. H&E stained images of major organs collected from untreated mice and mice after H-MnO_2-PEG/C&D-based combination therapy.

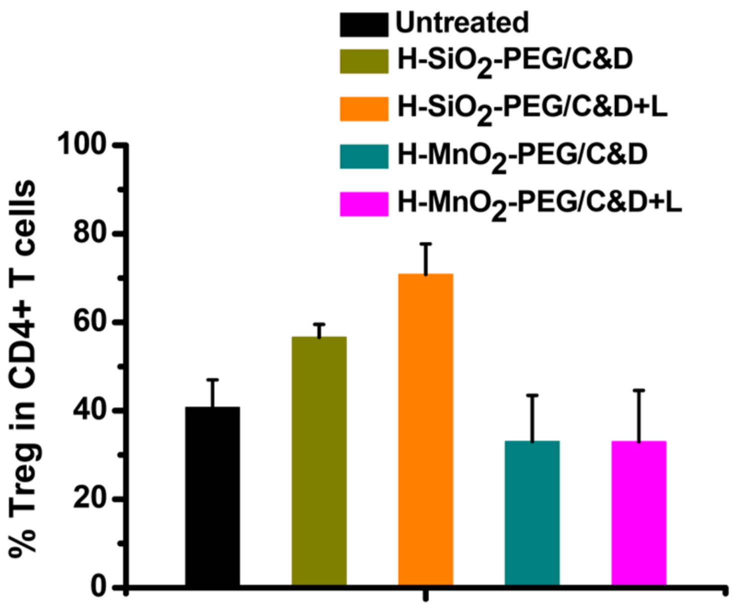

Supplementary Figure 10. Combined chemo-PDT treatment with H-MnO_2-PEG/C&D could slightly reduce the population of regulatory T cells (Treg) within tumors. Date are presented as means ± s.d. (n = 6).

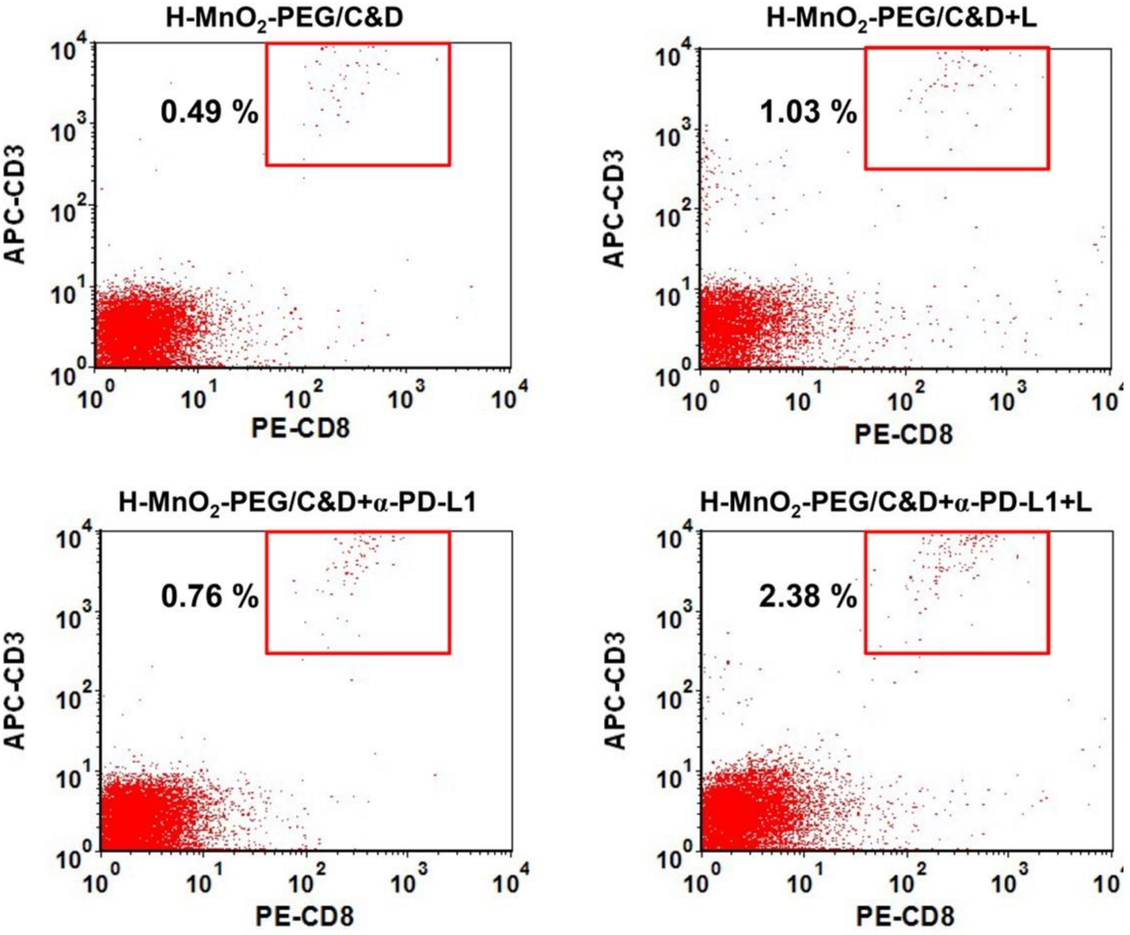

Supplementary Figure 11. Representative flow cytometry data of cytotoxic T lymphocytes (CTLs) infiltration in tumors. $CD3^+CD8^+$ cells were defined as CTLs.

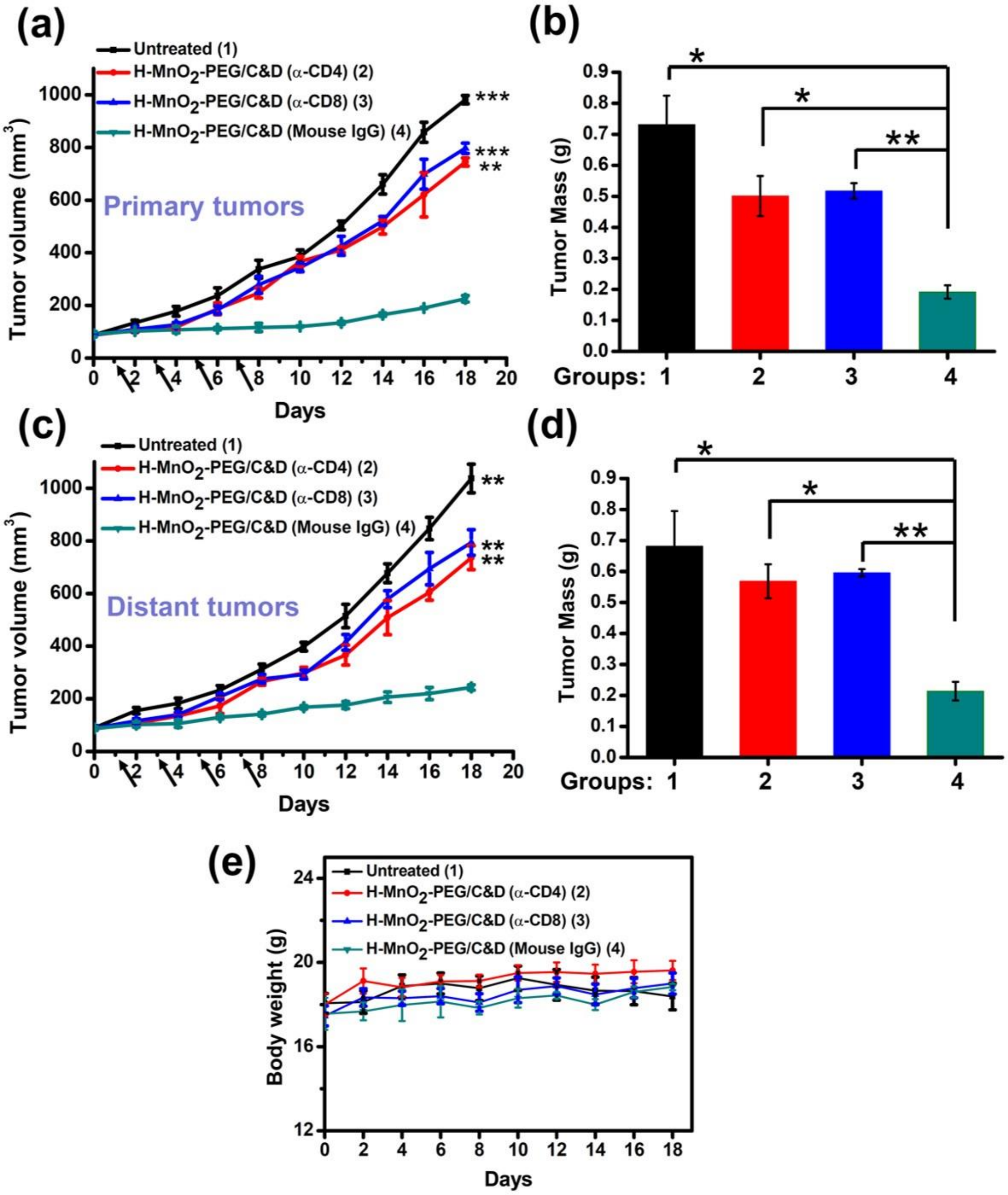

Supplementary Figure 12. (a&c) Growth curves of primary (a) and distant (c) tumors for different groups of mice after various treatments indicated. Error bars are based on the standard errors of the mean. The arrows represent the time points of anti-PD-L1 administration. Anti-CD4, anti-CD8, or mouse IgG were i.p. injected on day 0 and day 5 (200 μg/mouse for each dose). (6 mice per group). Average weights of primary (b) and distant (d) tumors collected from mice 18 d after initiation of various treatments. (e) Average body weights of mice after various treatments indicated. *P* values were calculated by the Tukey's post-test (***$p < 0.001$, **$p < 0.01$, or *$p < 0.05$) (Compared with untreated for (a&c)).
